# Cytokines and Signaling Molecules Predict Clinical Outcomes in Sepsis

**DOI:** 10.1371/journal.pone.0079207

**Published:** 2013-11-14

**Authors:** Christopher D. Fjell, Simone Thair, Joseph L. Hsu, Keith R. Walley, James A. Russell, John Boyd

**Affiliations:** 1 James Hogg Research Centre, University of British Columbia, Vancouver, British Columbia, Canada; 2 Pulmonary & Critical Care Medicine, Stanford University, Stanford, California, United States of America; University of Cincinnati, United States of America

## Abstract

**Introduction:**

Inflammatory response during sepsis is incompletely understood due to small sample sizes and variable timing of measurements following the onset of symptoms. The vasopressin in septic shock trial (VASST) compared the addition of vasopressin to norepinephrine alone in patients with septic shock. During this study plasma was collected and 39 cytokines measured in a 363 patients at both baseline (before treatment) and 24 hours. Clinical features relating to both underlying health and the acute organ dysfunction induced by the severe infection were collected during the first 28 days of admission.

**Hypothesis:**

Cluster analysis of cytokines identifies subgroups of patients at differing risk of death and organ failure.

**Methods:**

Circulating cytokines and other signaling molecules were measured using a Luminex multi-bead analyte detection system. Hierarchical clustering was performed on plasma values to create patient subgroups. Enrichment analysis identified clinical outcomes significantly different according to these chemically defined patient subgroups. Logistic regression was performed to assess the importance of cytokines for predicting patient subgroups.

**Results:**

Plasma levels at baseline produced three subgroups of patients, while 24 hour levels produced two subgroups. Using baseline cytokine data, one subgroup of 47 patients showed a high level of enrichment for severe septic shock, coagulopathy, renal failure, and risk of death. Using data at 24 hours, a larger subgroup of 81 patients that largely encompassed the 47 baseline subgroup patients had a similar enrichment profile. Measurement of two cytokines, IL2 and CSF2 and their product were sufficient to classify patients into these subgroups that defined clinical risks.

**Conclusions:**

A distinct pattern of cytokine levels measured early in the course of sepsis predicts disease outcome. Subpopulations of patients have differing clinical outcomes that can be predicted accurately from small numbers of cytokines. Design of clinical trials and interventions may benefit from consideration of cytokine levels.

## Introduction

Infection, the body's response (sepsis) and in extreme cases organ failure (septic shock) are complex inter-related processes with mortality rates exceeding 30% [1], [2]. Signaling compounds including cytokines, chemokines and growth factors mediate the immune response to infection, and are often highly elevated in patients with septic shock (reviewed recently by Rivers et al. [3]). The crucial early hours in sepsis determine the disease course [4]. When associated with organ failure and overwhelming shock this early phase is marked by an intense inflammatory response and disruption of the endocrine, coagulation, renal and cardio-pulmonary systems. In those who survive the intense pro-inflammatory phase of early septic shock, within days a compensatory anti-inflammatory response syndrome can develop. Mediated by inhibitors of the pro-inflammatory response this second phase is marked by a functional immune-suppression and high risk of secondary infection [5]. As the mediators of both pro and anti-inflammatory immune responses the pattern of cytokines have great promise as biomarkers of disease prognosis and for the discovery of novel therapeutics [5]–[7].

Despite this promise, data has often been derived from small cohorts and has been conflicting with respect to its prognostic value, possibly due to differing times in the disease process of biomarker measurement [3]. In particular, the most highly characterized pro- and anti-inflammatory cytokines, interleukin (IL)-6 and IL-10 respectively, were measured in patients with sepsis (the GenIMS study) and both had a positive correlation with mortality. This held true both during treatment for sepsis [8] and surprisingly after recovery from sepsis [9]. These and other similar results indicate that while studied in isolation, cytokines such as IL-6 and -10 levels correlate with harm in patients [10], [11] but protection in animal models [12]–[14]. When grouped together their roles may change significantly.

The complex interaction between the infection, patient cytokine/immune response and clinical outcomes requires large numbers of patients and sophisticated modeling methods to detect true patterns. This study uses plasma taken at standard times (at enrollment and 24 hours) in a very large group of patients enrolled into a randomized controlled study of vasopressin treatment in septic shock to measure 39 unique cytokines, chemokines and growth factors [1]. Using these measurements and employing hierarchical cluster analysis we defined groups of patients with highly similar cytokine responses. Clinical outcomes including mortality and duration of organ failures were then compared across cytokine-derived groups. Both upon enrollment and 24 hours we found a group of patients in whom the cytokine pattern was highly associated with both mortality and organ failure. We then compared the discriminatory ability of marker cytokines such as IL-2 versus the total 39. We have previously reported [15] the influence of vasopressin versus norepinephrine on mortality for this data set, and report here the broader implications of cytokine status on other clinical outcomes.

## Methods

### Subjects

The VASST study [1] was a multi-center randomized double-blind controlled trial of vasopressin versus norepinephrine in addition to standard vasopressors for the treatment of septic shock, Current Controlled Trials number ISRCTN94845869. Ethics approval was obtained for the VASST study from the University of British Columbia/Providence Health Care (UBC/PHC) Research Ethics Board on the 17th November 1999. Written informed consent was obtained from all patients, their next of kin, or another surrogate decision maker, as appropriate and approved by the UBC/PHC Research Ethics Board. The VASST study enrolled 778 patients who were greater than 16 years of age and had septic shock, as defined by the presence of two or more of the systemic inflammatory response syndrome (SIRS) criteria [15], proven or suspected infection, new dysfunction of at least one organ, and hypotension despite adequate fluid resuscitation (lack of response to 500 mL normal saline) and requiring vasopressor support of at least 5 µg/min of norepinephrine (or equivalent) for six hours [1]. Important exclusion criteria were unstable coronary syndromes, acute mesenteric ischemia, severe chronic heart disease (New York Heart Association class III and IV) and vasospastic diathesis [14]. Of these patients, 363 had blood plasma samples taken at baseline (enrollment at a median of 12 hours following admission to hospital) and 24 hours later.

### Cytokine assay

Plasma was stored at −80°C. In this study we included patients for whom we had both baseline and 24-hour plasma samples. A panel of 39 cytokines was measured in duplicate by Luminex MAG 39plex multiplex bead assay on a 100/200 System (Luminex Corporation, Austin, TX) according to the manufacturer's specifications. Positive and negative controls were assayed on each plate. The cytokines measured were (using both current and historical naming): CCL11 (Eotaxin), CCL2 (MCP1), CCL22 (MDC), CCL3 (MIP1a), CCL4 (MIP1B), CCL7 (MCP3), CD40LG (CD40Ligand), CSF2 (GMCSF), CSF3 (GCSF), CX3CL1 (Fractalkine), CXCL1 (GRO), CXCL10 (IP10), EGF (EGF), FGF2 (FGF2), FLT3LG (Flt3L), IFNA2 (IFNa2), IFNG (IFNG), IL10 (IL10), IL12B (IL12B), IL12P70 (IL12P70), IL13 (IL13), IL15 (IL15), IL17A (IL17), IL1A (IL1a), IL1B (IL1B), IL1RN (IL1RA), IL2 (IL2), IL2RA (IL2RA), IL3 (IL3), IL4 (IL4), IL5 (IL5), IL6 (IL6), IL7 (IL7), IL8 (IL8), IL9 (IL9), LTA (TNFB), TGFA (TGFa), TNF (TNFA), and VEGFA (VEGF). Values were reported by the assay system in pg/mL; these were converted to molar concentrations using Ensembl mature peptide amino acid sequences and molecular weights calculated using *pepstats* from the EMBOSS 4.1.0 software. Cytokine class or effect was taken from http://www.copewithcytokines.de/.

### Clustering

The *hclust* method (R statistical language package *stats*) was used for hierarchical clustering and visualization. The t-test for comparison of subgroups used Welch's method (*t.test* for unequal variance in R *stats* package) based on log10-transformed molar concentration cytokine values.

### Comparison of cytokine levels

Cytokine levels were compared using t-test with Welch's method (as above) with multiple comparison adjustment using false-discovery (FDR) rate of 0.05 (Benjamini-Hochberg method). Cytokine levels were log-transformed before comparison to make their distribution approximately normal. Cytokines with zero values were assigned to 1/2 the minimum cytokine level detected overall for any cytokine to allow log-transformation.

### Enrichment analysis

Enrichment (over-representation) of categorical features within clusters was assessed with p-values as probabilities from the hypergeometric distribution, which represents the likelihood of drawing items by chance of a particular feature in a set number of draws without replacement. All features (including survival at day 28 and 90) were examined for patient enrichment in clusters, and therefore multiple testing correction was applied using the false-discovery rate (FDR) correction of Benjamini-Hochberg (*p.adjust* method in R with parameter method *BH*). Enrichment of cytokines in cytokine clusters used pathway occupancy data from KEGG.db (R package version 2.8.0 by Marc Carlson).

### Survival analysis

Statistical differences of survival curves were assessed with the *survdiff* method of the R package *survival* from the chi-squared statistic of a G-rho rank test with *rho* = 0.

### Selection of marker cytokines

A minimal number of cytokines was selected to accurately place patients in the subgroups identified by the full panel of cytokines using logistic regression (*glm* function in R *stats* package with *family = “binomial”*). The full logistic regression equations were constructed starting with a single cytokine and adding additional cytokines until the resulting coefficients failed to be significant at p<0.05. We additionally considered products of cytokines when an adequate model was not constructed from any individual cytokines.

### Comparison of mortality to subgroup cytokine levels at uniform illness severity

Fisher exact test (*fisher.test* in R stats package, two-sided) was used to compare mortality of patients in each subgroup using a random sample of patients from each subgroup having the same APACHEII (Acute Physiology and Chronic Health Evaluation II) illness severity score. For each level of APACHEII score found in common all subgroups of patients, patients were randomly sampled from each subgroup for baseline and 24 hour data, to ensure the identical number of patients with each APACHEII score were present in each subgroup compared.

## Results

### Baseline characteristics

Characteristics of patients with cytokine measurements are shown in Table 1. These characteristics are statistically identical to those of the entire 778 patients from the original study with survival to 24 hours being the only restriction for the current study. As evidenced by a very high APACHE II severity of illness score (26 median score) as well as a high incidence of chronic organ failure these patients had a predicted mortality rate of approximately 50%. The site of infection was most commonly lung (44%), followed by abdominal (28%), genitourinary (5.1%) and intravascular (4.8%). Gram positive pathogens were slightly more common than gram negative (35% vs 20%), fungal organisms were the only cultured species in 18% of cases while in 24% of cases there were mixed organisms identified at the site of infection or via blood culture. Baseline characteristics of patients within each cytokine-derived subgroup (defined below) are shown in Table S7.

**Table 1:** Patient characteristics.

Characteristic	Value (% or IQR)
Age, years	63 (50–72)
Gender, Females	147 (40)
APACHEII score	26 (22–32)
Surgical Diagnosis	86 (24)
Severe septic shock	204 (56)
Vasopressin treatment	188 (52)
Activated Protein C treatment	61 (17)
Pre-existing conditions	
Chronic heart failure	73 (20)
Chronic lung disease	22 (6.1)
Chronic liver disease	42 (12)
Chronic renal failure	241 (66)
COPD	60 (17)
Cancer	68 (19)
Intravenous drug use	15 (4.1)
Alcoholism	54 (15)
Solid organ transplant	10 (2.8)
Recent trauma	20 (5.5)
Immuno-compromised	45 (12)
Chronic steroid use	69 (19)
Patient clinical measures at baseline	
Body temperature (°C)	38 (37–38)
Maximum heart rate (beats per minute)	128 (112–140)
MAP	72 (67–78)
WBC	14 (7.8–21.1)
Platelets	167 (89–257)
Pao2Fio2	194 (143–260)
Creatinine	155 (94–254)
Pathogen	
Gram positive only	76 (35)
Gram negative only	44 (20)
Fungus only	38 (18)
Virus only	2 (1)
Mixed infection	52 (24)
Initial site of infection	
Lung	155 (44)
Intravascular	17 (4.8)
Abdomen	99 (28)
Skin	32 (9)
Genitourinary	18 (5.1)
CNS	2 (0.56)
Bone/joint	5 (1.4)
Other site	28 (7.9)

### Patient cytokine-derived subgroups

**Figure 1:**
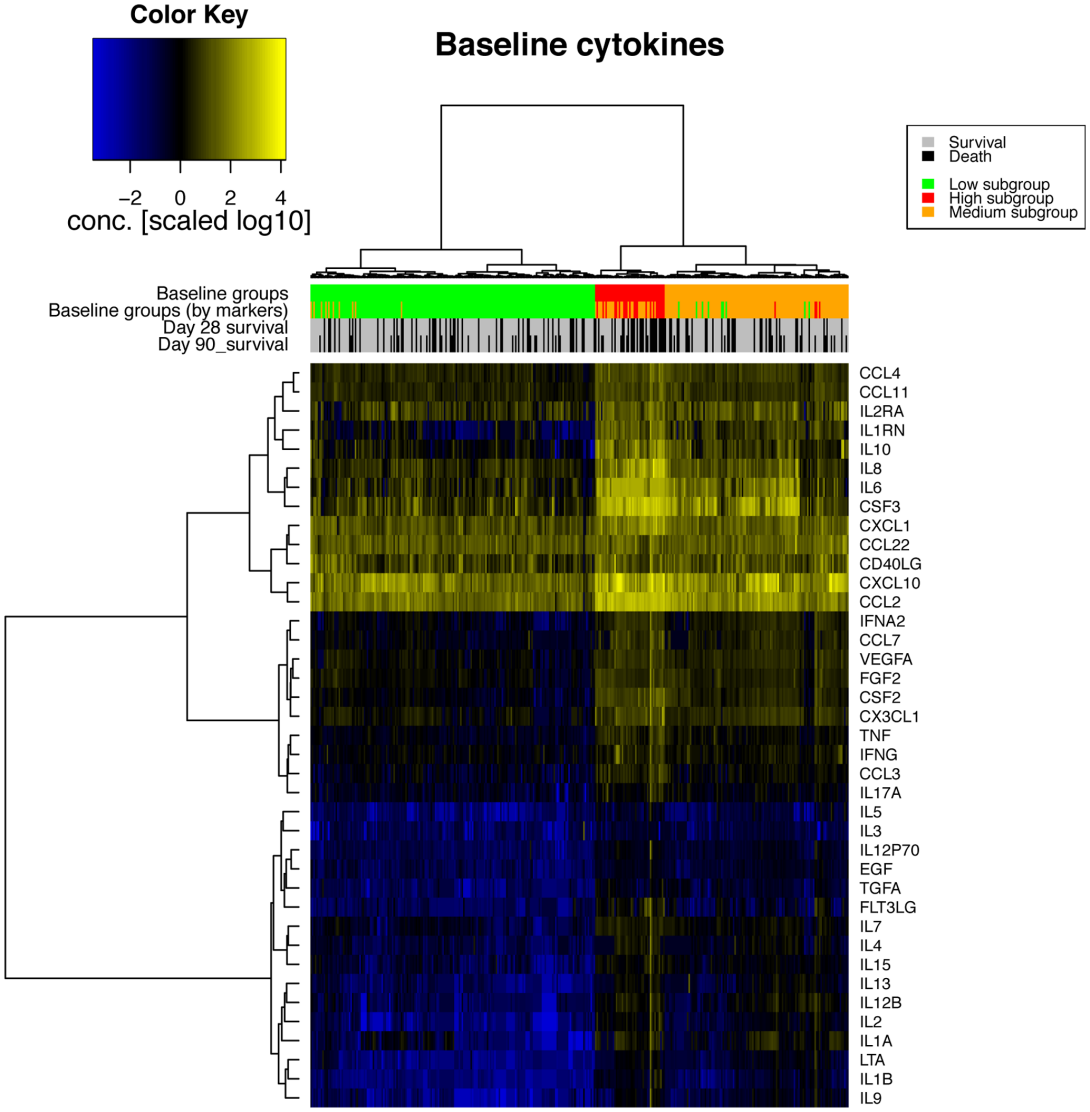
Levels of signaling molecules at baseline. Patient subgrouping, survival and features are indicated on the top colored rows. The *Baseline groups* are the Low, Medium and High subgroups using baseline cytokines and all 39 signaling molecules. The *Baseline groups (by markers)* are predicted groups using IL2 and CSF2 cytokines only.

**Figure 2:**
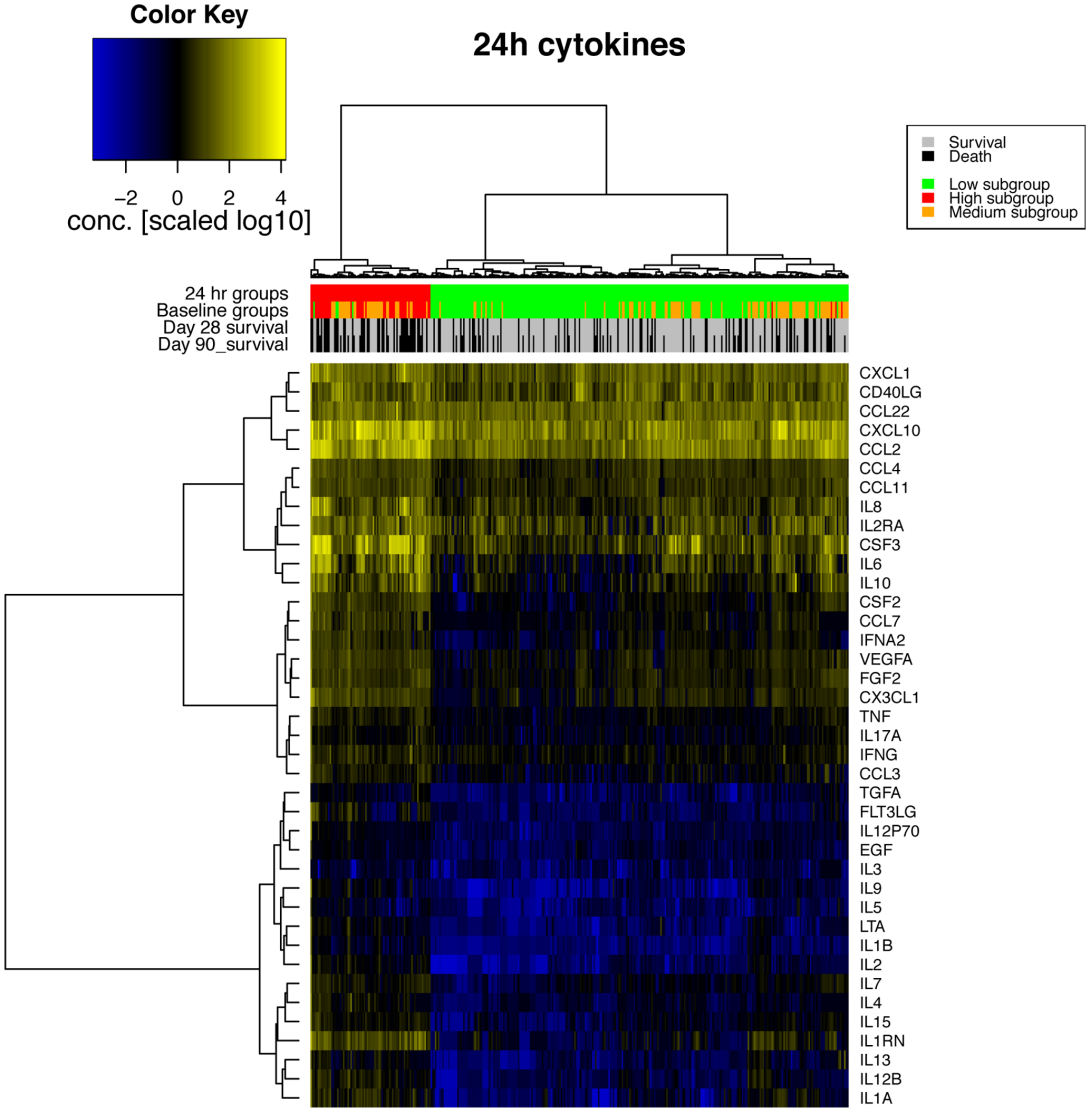
Levels of signaling molecules at 24 hours. Patient subgrouping, survival and features are indicated on the top colored rows. The *24 hr groups* are the Low and High subgroups based on 24 hour cytokines. The *Baseline groups* are the corresponding subgroups the patients are in using baseline data.

We used an assay measuring a variety of different signaling molecules including cytokines, growth factors and chemokines (we refer to these as “cytokines” for brevity except where this creates confusion) from undiluted serum, with two freeze thaw cycles. All samples were treated identically regardless of the patient location or outcome. Approximately 20% of cytokine values were below detectable limits, and 0.4% were above limits. Where limits were reported, the measured value was taken as the limit (e.g. where the assay value is reported as >2.55 or <2.55, for calculations the value was 2.55). Where assay values were zero, values of ½ the lowest reported value of any cytokine was used to allow for logarithmic transformation. Cytokine levels were used to cluster patients (horizontally in Figures 1 and 2) into subgroups having similar expression. Based on cytokines levels at baseline, three subgroups of patients can be observed (Figure 1). (Below, we refer to these as the Low, Medium and High cytokine subgroups to reflect the overall cytokine levels.) Of this grouping, the leftmost (Low subgroup) in Figure 1 (192 patients) showed lowest overall level of cytokines, the rightmost (Medium subgroup) containing 124 patients showed higher cytokine levels, and the middle subgroup (High subgroup) shows highest overall levels of cytokines. Using cytokines measured at 24 hours, two subgroups are apparent (Figure 2), with a high cytokine subgroup of 81 patients, and a Low cytokine subgroup with 282 patients. Of the 47 patients in the High subgroup at baseline, 43 (91%) were also in the corresponding High subgroup using cytokines sampled at 24 hours. The Medium subgroup defined using baseline The clustering of patients into subgroups was performed based on cytokine levels alone. We went on to examine the patient features that were enriched in subgroups as this may reflect either the cause or effect of the underlying biology of cytokine patterns. All 39 cytokines are significantly different between subgroups of patients (p<0.03) (see Table 2 and Table 3 for cytokines with highest differences; see Table S1 and Table S2 for all cytokines). The number of patients receiving the VASST study drug, vasopressin, did not differ between subgroups using baseline or 24 hour cytokine data (p>0.8).

**Table 2:** Cytokine levels at baseline in patient subgroups 1 to 3.

Cytokine	Cytokine cluster	Low Patient Subgroup	High Patient Subgroup	Medium Patient Subgroup	Ratio Patient Subgroups High to Low	p-value (Patient Subgroups High to Low)	Ratio Patient Subgroups High to Medium	p-value (Patient Subgroups High to Medium)
IL6	4	2 (0.89–4.7)	420 (380–430)	26 (5.4–70)	213.6	6.9E-49	16	2.5E-21
CSF3	4	3.4 (1.7–9.6)	640 (450–950)	57 (8.4–340)	185.9	6.1E-44	11.2	7.4E-16
IL1RN	4	0.16 (0.031–0.7)	22 (11–42)	4.9 (2.2–8)	135.7	2.6E-48	4.5	3.4E-13
IL10	4	0.66 (0.31–1.7)	58 (19–140)	4.2 (1.5–12)	87.1	1.2E-29	13.8	1.2E-13
IL9	1	0.0063 (0.0019–0.033)	0.38 (0.096–0.59)	0.16 (0.095–0.3)	60.1	1.8E-19	2.4	1.5E-01
LTA	1	0.0076 (0.0063–0.022)	0.4 (0.11–0.68)	0.16 (0.082–0.33)	52.6	2.1E-29	2.5	1.2E-03
IL1A	1	0.026 (0.0086–0.2)	1.4 (0.63–3)	0.49 (0.13–1.8)	52.2	5.7E-23	2.8	2.9E-03
IL8	4	2.7 (1.5–4.6)	140 (34–560)	9.3 (5–21)	50.9	8.4E-23	14.7	9.2E-15
IL2	1	0.015 (0.0017–0.028)	0.64 (0.43–1.1)	0.2 (0.083–0.44)	41.8	3.6E-44	3.1	2.1E-10
FLT3LG	1	0.025 (0.014–0.073)	0.93 (0.53–2.3)	0.12 (0.025–0.39)	37.7	1.0E-22	7.7	4.9E-13
IL1B	1	0.0088 (0.0043–0.016)	0.28 (0.2–0.5)	0.078 (0.032–0.16)	32.2	2.0E-30	3.6	1.4E-11
IL12B	1	0.036 (0.019–0.086)	1.1 (0.66–1.7)	0.53 (0.19–1)	30.1	2.5E-36	2	9.9E-07
IL13	1	0.024 (0.012–0.086)	0.66 (0.2–1.1)	0.24 (0.12–0.57)	27.5	1.2E-30	2.7	1.8E-04
CCL2	3	28 (17–54)	710 (520–910)	120 (56–210)	25.5	1.2E-55	6.1	2.2E-30
CSF2	2	0.38 (0.21–0.63)	7.5 (5.3–12)	2.2 (1.2–3.5)	19.4	5.2E-35	3.4	3.4E-15
IL4	1	0.07 (0.022–0.17)	1.1 (0.18–1.9)	0.33 (0.18–0.67)	16.1	6.6E-18	3.4	1.7E-04
CCL7	2	0.26 (0.17–0.6)	3.8 (2.2–6.8)	1.7 (0.99–2.8)	14.6	2.2E-16	2.3	6.7E-05
CXCL10	3	40 (19–110)	510 (180–1000)	110 (49–400)	12.8	4.1E-15	4.6	4.1E-05
IL15	1	0.069 (0.013–0.18)	0.88 (0.52–1.4)	0.44 (0.26–0.71)	12.6	8.9E-34	2	1.1E-07
IL7	1	0.14 (0.042–0.31)	1.7 (1.3–2.4)	0.69 (0.43–1.1)	11.7	1.2E-43	2.4	3.1E-13
IFNA2	2	0.33 (0.13–0.69)	3.7 (2.5–4.7)	1.7 (0.93–2.7)	11.4	2.6E-33	2.2	3.6E-07
TNF	2	0.26 (0.16–0.46)	2.9 (1.8–5.3)	0.85 (0.54–1.4)	11.1	2.9E-24	3.4	7.0E-12

Concentrations of cytokines are shown for those having highest ratio between patient groups. Values are median, and interquartile ranges in pM. Ratio is the ratio of corresponding medians. The p-value is from t-test on log10-transformed cytokine values adjusted for multiple testing. See Table S1 for full list.

**Table 3:** Cytokine levels at 24

Cytokine	Cytokine Subgroup	High Patient Subgroup	Low Patient Subgroup	Ratio	p-value
IL1RN	1	5.5 (2.4–13)	0.14 (0.03–0.66)	38.9	2.8E-53
CSF3	3	100 (9.8–720)	3 (1.4–8.2)	35.0	9.6E-21
IL1A	1	1 (0.44–2.4)	0.033 (0.013–0.16)	31.1	5.7E-38
IL9	1	0.21 (0.096–0.41)	0.0094 (0.002–0.051)	22.2	1.9E-28
IL6	3	20 (6–120)	0.94 (0.21–3.7)	21.8	5.2E-22
IL2	1	0.39 (0.19–0.62)	0.018 (0.0034–0.051)	21.6	5.0E-52
LTA	1	0.23 (0.11–0.46)	0.012 (0.0063–0.05)	19.6	6.7E-41
FLT3LG	1	0.47 (0.068–1.1)	0.025 (0.014–0.073)	19.3	1.3E-13
IL12B	1	0.74 (0.35–1.4)	0.041 (0.021–0.086)	18.1	7.7E-40
IL1B	1	0.13 (0.074–0.29)	0.0088 (0.0046–0.021)	15.0	1.2E-32
IL10	3	9.8 (2.7–27)	0.66 (0.24–1.8)	14.9	3.5E-25

Concentrations of cytokines are shown for those having highest ratio between patient subgroups. Values are median, and interquartile ranges in pM. Ratio is the ratio of medians of High and Low cytokine subgroup values. The p-value is from t-test on log10-transformed cytokine values adjusted for multiple testing. See Table S2 for full list.

### High cytokine subgroups predict mortality independently of clinical severity scoring

**Figure 3:**
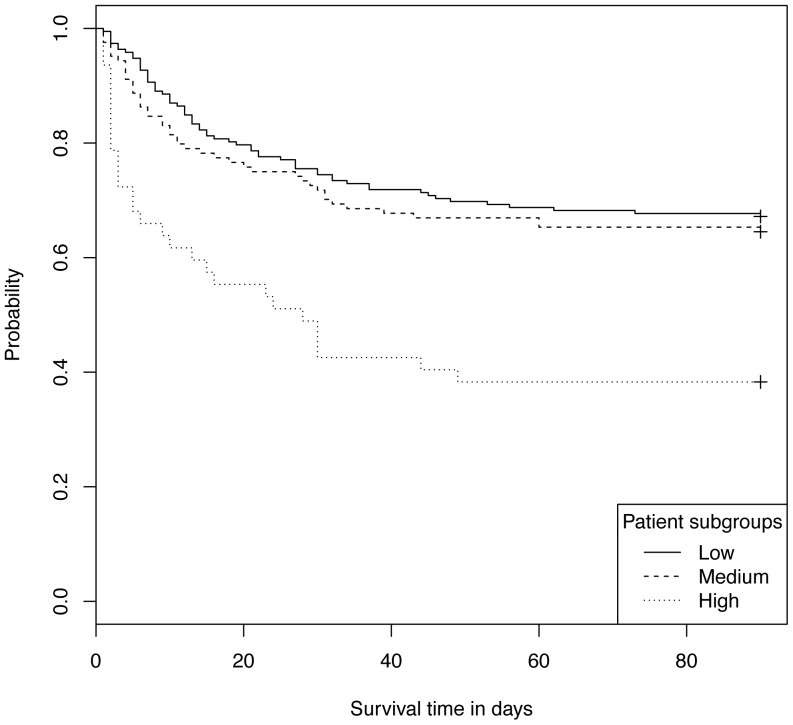
Survival curves for patients in Low, Medium and High subgroups using cytokines at baseline. P-values<0.001 for difference in curves between Low and High as well as Medium and High , but not significant between Low and Medium.

**Figure 4:**
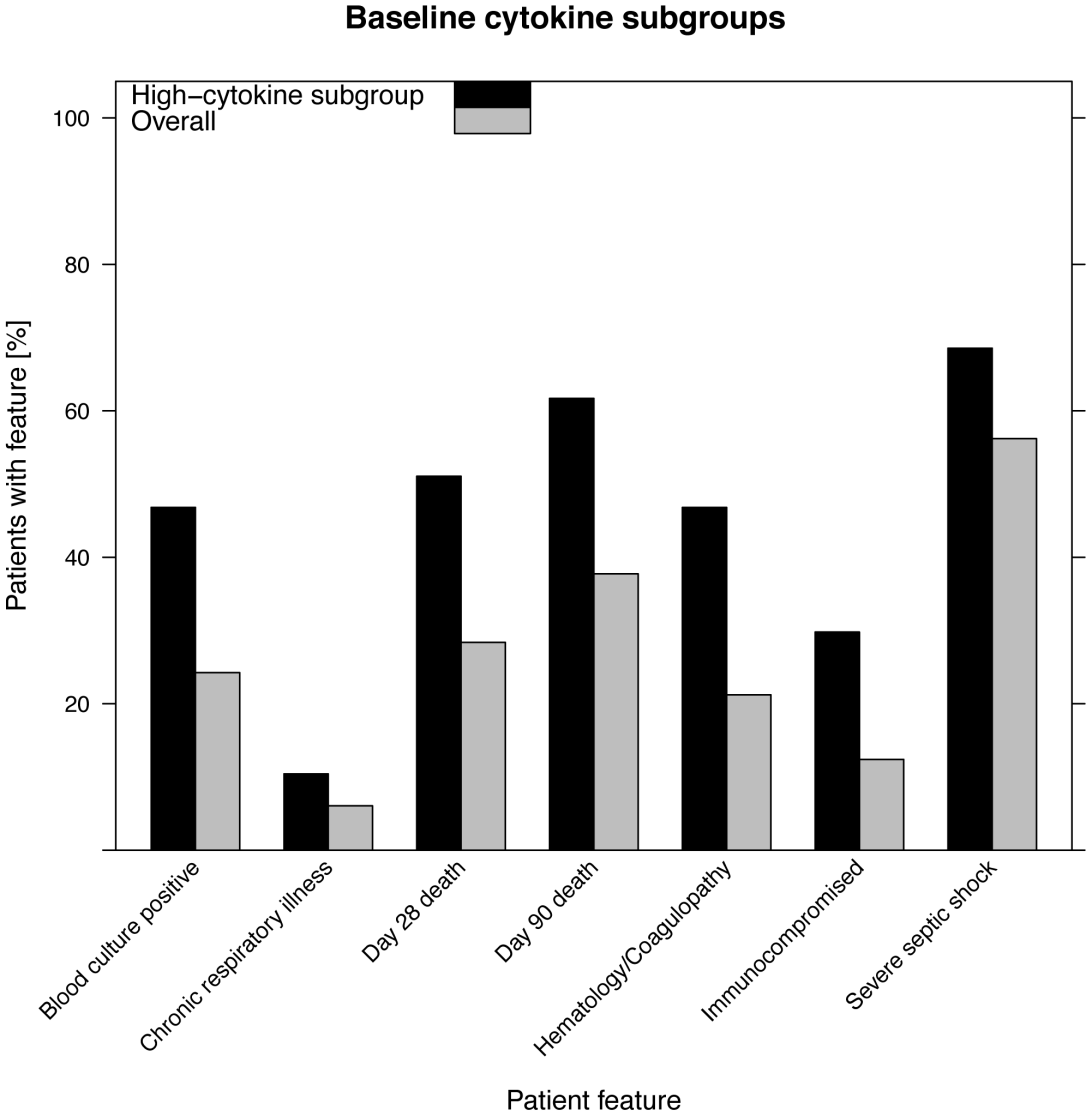
Features of patients enriched in High subgroup using cytokines at baseline. Enrichments are all significant at p<0.05, adjusted for multiple testing. The comparison was to overall (entire) patient population in the study.

Using baseline cytokine levels, patients in the High subgroup show dramatically increased risk of death at all time points: a relative increase of 80% at day 28 (51% mortality High patients vs. 28% mortality overall) and relative increase of 63% at day 90 (62% vs. 38% mortality). The survival curves for the three subgroups are shown in Figure 3; Low and Medium subgroups have similar profiles, while the survival rate for the High subgroup is much lower. Consistent with survival outcomes, the high-cytokine subgroup was significantly enriched for conditions associated with poor outcome: severe septic shock (defined as patient requiring >15 µg/min of the vasopressor norepinephrine), a positive blood culture, and organ failure involving hematology or coagulation (Figure 4) (see Table S3 for details). Interestingly, despite higher cytokine levels, High subgroup patients were more likely to be chronically immunocompromised (Figure 4). Chronic respiratory illness is associated with the lowest levels of overall cytokines (reduced by 37% in Low subgroup) while Medium subgroup is enriched in severe septic shock by 22%).

**Figure 5:**
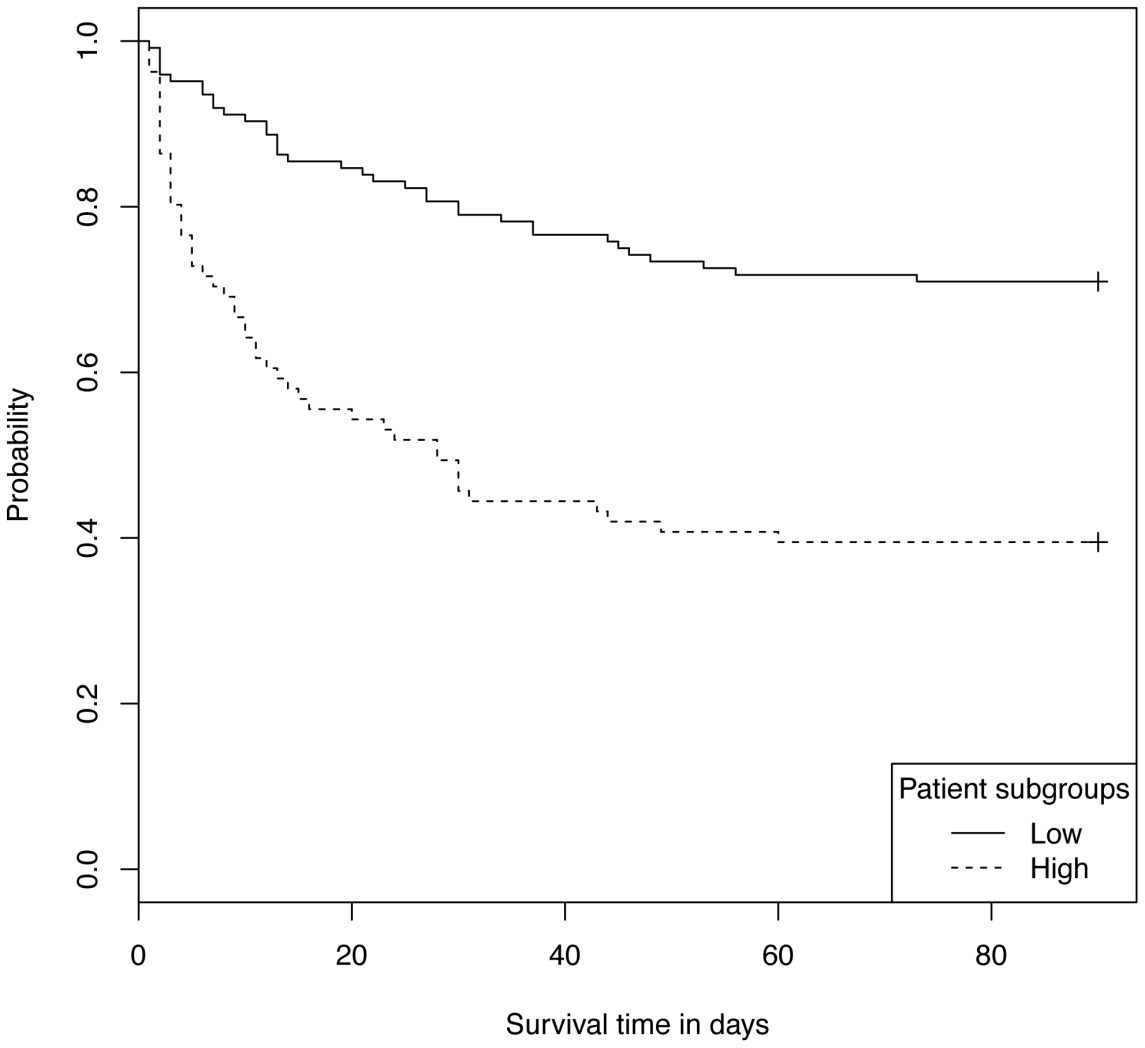
Survival curves for patients in Low and High subgroups using cytokines at 24 hours. P-value<0.001 for difference in curves between Low and High.

**Figure 6:**
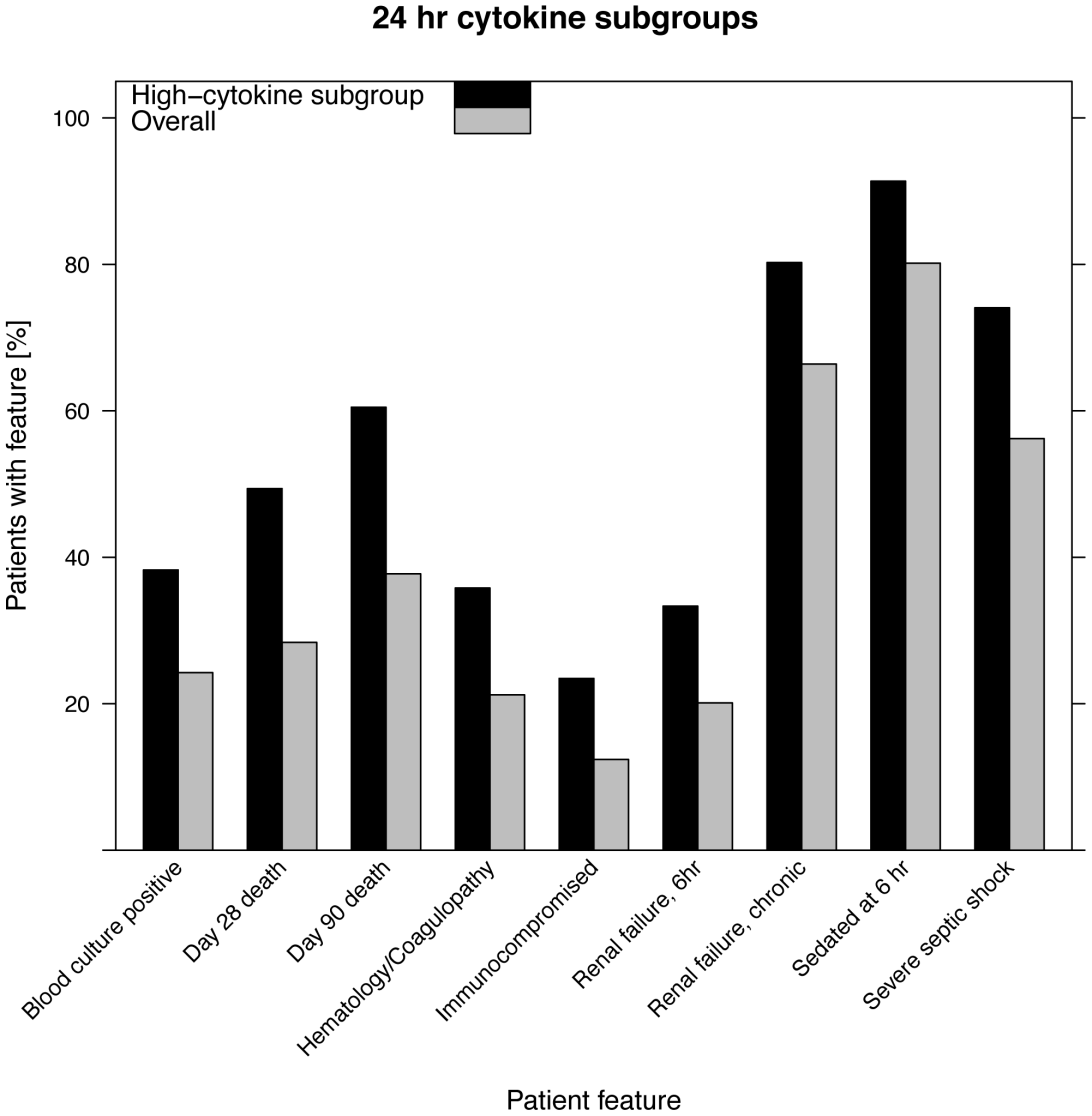
Features of patients enriched in High subgroup using cytokines at 24 hours. Enrichments are all significant at p<0.05, adjusted for multiple testing. Comparison was to overall (entire) patient population in the study.

As for the High subgroup based on baseline samples, the High cytokine based on cytokines measured at 24 hour has poorer survival (Figure 5) and significantly higher rates of renal failure (both chronic and acute renal failure at 6 hours), positive blood culture, chronically immunocompromised, severe septic shock, and need for sedation at 6 hours (Figure 6, and see Table S4 for details).

### Cytokine clusters

**Figure 7:**
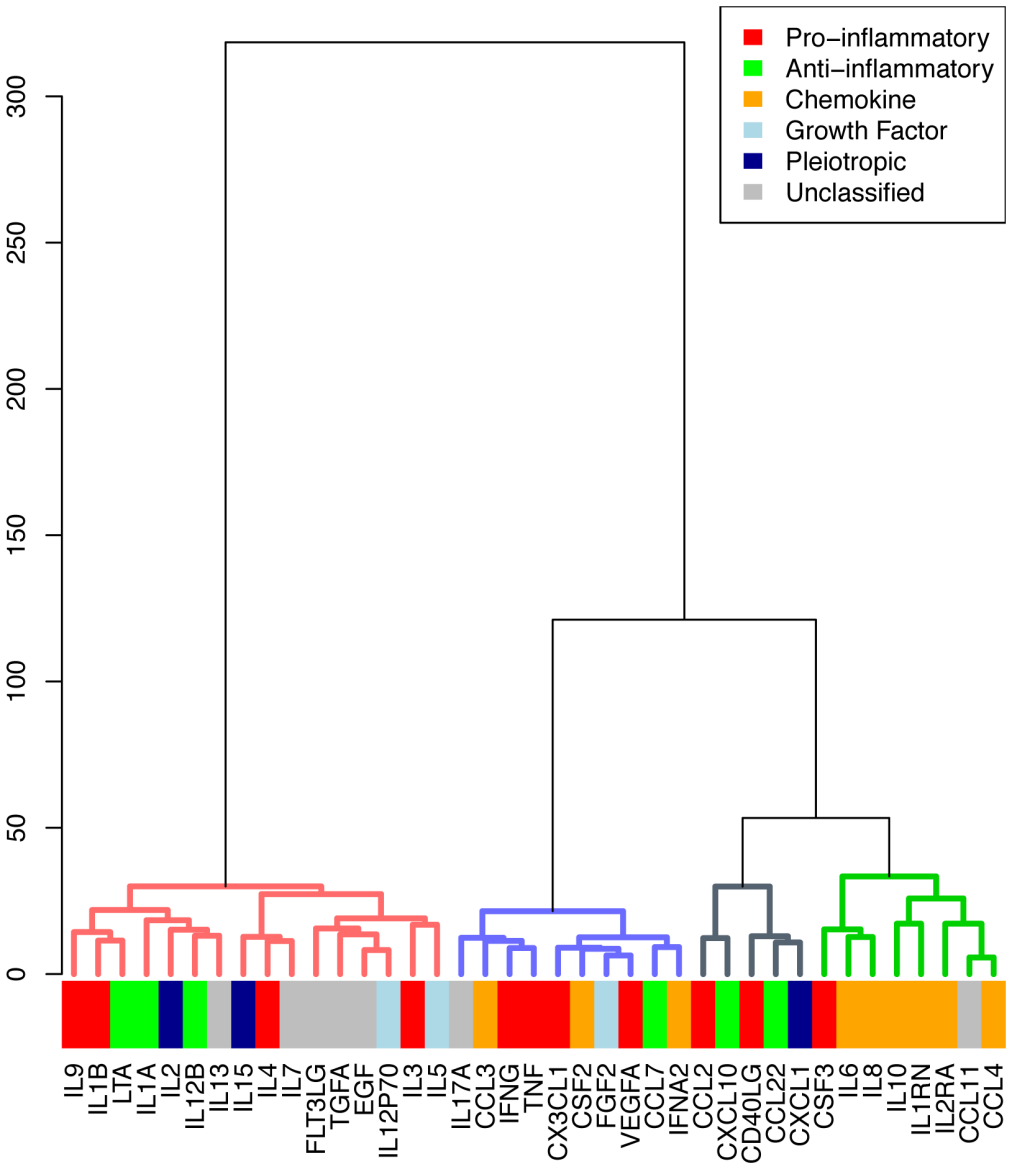
Clustering of signaling molecules measured at baseline. Colors indicate types of signaling molecules.

**Figure 8:**
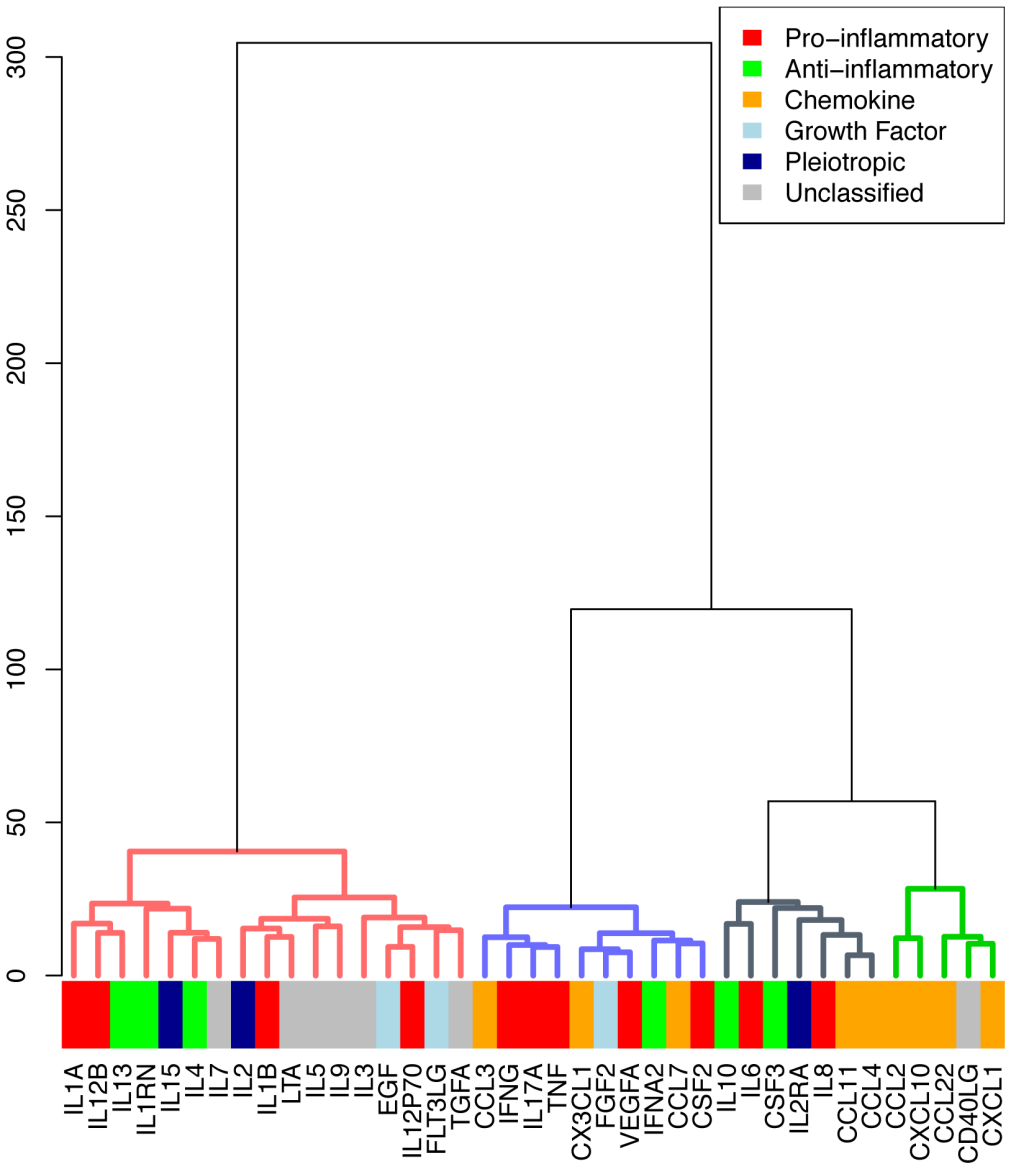
Clustering of signaling molecules measured at 24 hours. Colors indicate types of signaling molecules.

In addition to clustering patients, cytokines were also clustered based on variation of cytokines across patients (indicated by the vertical dendrogram on the left hand-side of each heatmap). We observed that the cytokines do not cluster together significantly by type, except for chemokines and unclassified cytokines (see Figures 7 and 8). Of the cytokines involved in TH2 response, IL4, IL5, IL9 and IL13, these all cluster together both at baseline and 24 hours. Enrichment for KEGG pathways was not significant after adjusting for multiple comparisons. However, we report results for unadjusted p-values (<0.05) since the penalty for multiple testing may be excessive. Cytokines in three clusters with data from 24 hours are reported: cluster 1 (Chemokine signaling pathway, KEGGID 04062, p = 0.01, p-adjusted = 0.8), cluster 2 (Malaria, KEGGID 05144, p = 0.04, p-adjusted = 1.0), and cluster 3 (Natural killer cell mediated cytotoxicity, KEGGID 04650, p = 0.003, p-adjusted = 0.18). Cytokines in cluster 4 had best enrichment for Type I diabetes mellitus (KEGGID 04940, p = 0.09, p-adjusted = 1.0). The same pathways are identified using baseline cytokines. No p-value was significant after correcting for multiple testing across all pathways (all p>0.18) (see Table S5 for details).

Some patients transitioned from a different subgroup defined using baseline data compared to 24 hour data (Table 4). To determine whether the subgrouping based on baseline or 24 hour data were more significantly associated with mortality, we compared the survival curves of the patients who transitioned between subgroups to the survival curves of the baseline subgroupings and 24 hour subgrouping. The majority of patients stayed in similar cytokine subgroups: most Low and Medium subgroup patients at baseline were in the Low subgroup at 24 hours. Nearly all High subgroup patients at baseline were also High at 24 hours: 4 patients transitioned from High to Low, and 4 patients from Low to High. The mortality curve was significantly different for the Low to High patients, compared to Low baseline (p = 0.007) showing these patients had mortality risk similar to the High subgroup. In addition, 34 patients transitioned from Medium subgroup at baseline to High at 24 hours; these patients had survival curves significantly different from the overall Medium baseline subgroup (p = 0.02), and similar to the High at 24 hours subgroup.

**Table 4:** Patient subgroup transitions.

Baseline patient subgroup	24 hour patient subgroup	Number	90 day mortality, %	p-value compared baseline	p-value compared 24 h
Low	Low	188	60 (32)	0.8	0.8
Medium	Low	90	25 (28)	0.2	0.6
High	Low	4	2 (50)	0.8	0.2
Low	High	4	3 (75)	0.007*	0.5
Medium	High	34	19 (56)	0.02*	0.6
High	High	43	27 (63)	0.9	0.8

Numbers of patients found in each combination of subgroups based on baseline cytokines and 24 hr cytokines. Mortality at 28 days is reported and p-value for the difference between the survival curve for the patients transition versus the survival curve of the subgroups based on the baseline and 24 hr cytokines.

### Discriminatory characteristics of marker cytokines versus all cytokines

We were interested in determining a smaller set of cytokines that was as effective as the entire panel for the prediction of clinical risk. For this analysis, we used logistic regression to predict the patient subgroup. We initially used multilevel logistic regression to predict the three subgroups for baseline data as a single calculation but found presentation of the results cumbersome. Therefore we present the two-level models (in-the-subgroup vs not-in-the-subgroup) for each of the 3 subgroups found for baseline data, in turn. We iteratively added cytokines to the logistic regression relationship until additional coefficients produced non-significant coefficients at p<0.05. (See Table S6 for complete model coefficients.) Using the baseline cytokine data, the Low cytokine subgroup could be predicted well (area under the receiver operating curve, AUC = 0.96) using a single cytokine, IL2; as well, the High subgroup could be predicted well with CSF2 (AUC = 0.98) (Table 5).

**Table 5:** Logistic regression results for predicting patient subgroup with single cytokine models.

Cytokine time	Patient subgroup	Intercept	Signaling molecule, Coefficient	AUC
Baseline	Low	2.21±0.23 (<2e-16)	IL2, −1.91±0.24e+13 (9e-16)	0.96
Baseline	Medium	−0.66±0.11 (3e-09)	LTA, 2.2±5.9e+10 **(0.707)**	0.82
Baseline	High	−4.91±0.53 (<2e-16)	CSF2, 8.0±1.1e+11 (6e-14)	0.98
24 hours	High	−2.82±0.24 <2e-16	IL2, 9.3±1.1e+12 (<2e-16)	0.95

Coefficients are reported with standard errors and p-values for coefficients being non-zero in round brackets.

However, the best performance for the Medium subgroup using an additive model of signaling molecules gave LTA alone, but resulted in non-significant model coefficient but a significant AUC ( = 0.82, Table 5). A permutation test (by calculating AUC after randomizing Medium subgroup labels, performed 100 times: mean 0.57, standard deviation 0.2, maximum 0.65)) showed this level of AUC had an empirical p-value<0.01 despite non-significant model coefficient. Both IL2 and CSF2 (which gave highly-significant logistic regression coefficients for Low and High subgroups) gave poorer AUC values than LTA (0.79 and 0.76 respectively), and non-significant coefficient p-values (p>0.4). We examined other forms of the models to investigate the discrepancy between high performance for Low and High cytokine subgroup models and poor performance for Medium subgroup. Models using the product of IL2 and CSF2 (IL2×CSF2) alone, or terms for IL2×CSF2 in addition to CSF2 similarly did not produce models with significant coefficients. However, terms for IL2 in addition to IL2×CSF2 produced highly significant models with coefficient p-values<1e-4. The presence of the IL2 term also allowed a significant term for CSF2 (Table 6), resulting in AUC of 0.84. Using cytokines from 24 hours, the two patient subgroups were well identified with IL2 (AUC of 0.95) or IL2×CSF2 (AUC = 0.99), see Table 6. As can be seen in Figure 1 (colored line annotated “Baseline groups (by markers)”, the High and Medium subgroups are not well distinguished using this small subset of markers.

**Table 6:** Logistic regression results for predicting patient subgroup with cytokine product models.

Cytokine time	Patient subgroup	Intercept	Signaling molecule, Coefficient	AUC
Baseline	Low	2.21±0.23(2e-16)	IL2×CSF2, −1.52±0.21e+25 (1e-12)	0.98
Baseline	Medium	−1.34±0.17(9e-15)	IL2×CSF2, −4.00±0.99e+23 (5e-05)IL2, 3.92±0.71e+12 (4e-08)CSF2, 1.18±0.41e+11 (0.00473)	0.84
Baseline	High	−4.91±0.53 (<2e-16)	CSF2, 8.0±1.1e+11 (6e-14)	0.98
24 hours	High	−3.43±0.32 (<2e-16)	IL2×CSF2, 9.5±1.3e+24 (3e-13)	0.99

Coefficients are reported with standard errors and p-values for coefficients being non-zero in round brackets.

### Subgroups of patients defined by cytokines predict mortality independent of illness severity

A sample of patients from each subgroup was chosen at each level of APACHEII illness severity score encountered in all subgroups, repeated 100 times. Mortality at day 28 was compared to the patient subgroup (Low, Medium, and High with baseline data; and Low and High with 24 hour data). Mortality for these patients is shown in Table 7. Due to small sample sizes, comparisons of baseline groups is not significant at 0.05 (median 0.07, 95% CI 0.006–0.5); however, for all 100 random samples comparisons of Low to High subgroup at baseline, the odds ratio of mortality is always <0.74 (median 0.39, 95% CI 0.24–0.67). Significance and odds ratio for Medium baseline subgroup versus High subgroup similarly trends in the same direction but lacks power due to low numbers. Using cytokines at 24 hours, mortality differs significantly between patient subgroups compared to High subgroup (odds ratio median 0.34, CI 95% 0.23–0.43, p<0.01 in all samples).

**Table 7:** Mortality of patients by subgroup with constant illness severity.

Cytokine time	Number of patients per subgroup	APACHEII score [median (min – max)]	Patient subgroup	Mortality Odds Ratio [median, 95% CI]	p-value compared to High subgroup [median, 95% CI]
Baseline	39	26 (17–39)	Low	0.39 (0.24–0.67)	0.07 (0.006–0.5)
Baseline	39	26 (17–39)	Medium	0.54 (0.35–0.82)	0.3 (0.04–0.8)
Baseline	39	26 (17–39)	Low+Medium	0.48 (0.36–0.63)	0.077 (0.015–0.25)
24 hours	76	26 (17–43)	Low	0.34 (0.23–0.43)	0.002 (3e-05-0.01)

The mortality of patients in each subgroup with constant APACHEII score is shown with p-value using Fisher's exact test. Mortality odds ratio is the comparison subgroup versus the High subgroup.

## Discussion

The most significant finding from this work is that distinct subgroups of patients with septic shock exist and can be identified from patterns of signaling molecules such as cytokines, chemokines and growth factors measured in plasma. Three subgroups of patients are identified from clustering analysis of samples taken before treatment or within hours of initiation of treatment (the baseline measurements). The patient subgroup with highest cytokine levels showed significantly higher mortality as well as other attributes of more severe disease. In contrast, the patients with low and medium levels of cytokines (which we termed Low and Medium subgroups) have similar mortality. Using samples taken 24 hours after initiation of treatment, clustering analysis identifies two subgroups, again where high cytokine levels are associated with worse outcomes. Most of the patients who grouped into Low or High subgroups using baseline data were also found in the Low and High subgroups respectively using 24 hour data. However, four patients were in the Low baseline subgroup and 34 were in the Medium subgroup at baseline but were in the High subgroup at 24 hours. These patients had mortality similar to High subgroup patients. While the Medium subgroup at baseline had a similar 90-day mortality (35%) compared to Low baseline subgroup (33%), these 34 patients who clustered with the High subgroup at 24 hours, had significantly higher 90-day mortality (56%), comparable to the High subgroup at baseline (62%) and at 24 hours (60%). The significance is not clear of these transitions from lower mortality subgroup defined at baseline to higher mortality subgroup at 24 hours; this may represent opportunity for a clinical intervention to customize treatment for this small group of patients, or may be an artifact of these analysis methods.

The signaling molecules were also clustered based on variation across patients to form clusters of molecules. These did not cluster significantly based on type, such as traditional classifications such as pro-inflammatory and anti-inflammatory. As the reported biological effects of cytokines do not necessarily correspond to their regulation (transcriptional or otherwise), it is perhaps not surprising that cytokines with similar roles did not cluster together. More surprising is that cytokines classically described as NFkB-regulated early response such as IL-6 and TNF alpha do not cluster together. Less studied in septic shock are the TH2 cytokines IL4, IL5, IL9 and IL13. Interestingly these all cluster together both at baseline and 24 hours.

We were interested in determining whether a small number of cytokines was as effective as the entire panel for the prediction of clinical risk. For this purpose we constructed logistic regression models with a small number of terms to predict the patient subgroup that was originally calculated using all 39 signaling molecules. To gain more insight into model performance, we chose to model the three subgroups for baseline data by predicting each subgroup separately (e.g. distinguish Low subgroup patients from combined Medium+High subgroups). For the Low and High patient subgroups, good performance was achieved with single cytokines (IL2 and CSF2 separately, respectively) as assessed by the area under the ROC curve (AUC, 0.96 and 0.98) and the coefficient significance p-value, though the product of IL2 and CSF2 was superior (AUC and 0.98 and 0.99). Signs on the regression models indicate that low levels of IL2 predicts Low patient subgroup; and high CSF2 predicts High patient subgroup.

However, the Medium subgroup was predicted relatively poorly with a single cytokine, (LTA, lymphotoxin alpha), with poor coefficient significance (p-value = 0.7) indicating a poor fit to the data. However, we found that a model producing a good fit was possible when the product of IL2 and CSF2 concentrations was included in addition to the terms for IL2 and CSF2. Interestingly, the coefficient on the interaction term of the product of IL2 and CSF2 has reversed sign compared to the terms for IL2 and CSF2 (Table 6). Thus, the best prediction for the Medium patient subgroup demonstrates an interaction between levels of different cytokines that may suggest regulatory behaviour in these patients, with some patients transitioning at 24 hours to a High cytokine profile (with associated high mortality) and some transitioning to a Low cytokine profile (associated with lower mortality). We note that levels of cytokines were highly correlated as can be observed in the heatmap patters of Figures 1 and 2. Therefore, other cytokines with similar expression patterns may work equally well. Increasing the complexity of models based on marker cytokines increase accuracy of subgroup prediction (for example, Table S6) at the cost of complexity and difficulty of interpretation. With such highly correlated data as cytokines, assessing complex machine learning using methods such as cross-validation presents difficulties which we will address in future work.

We were interested in determining if the cytokine subgroups add additional information for predicting patient outcome not already available from clinical features. We compared mortality of samples of patients with identical APACHEII illness severity score from each subgroup (Table 7). In all comparisons between subgroups for the same time point, the odds ratio is <1 for Low or Medium patient subgroups. However, due to the small numbers of patients available for these matched samples at baseline, median p-value obtained from 100 sampling iteraction is >0.05. Using the larger two subgroups from 24 hour cytokines, the mortality odds ratio compared to High subgroup of 0.34 is significant (median p-value 0.002). Thus, the cytokine data add additional information on patient outcome not available from clinical scoring: the cytokine profiles are not merely indicative of more severe illness; for the same severity of illness, higher cytokines indicate higher likelihood of non-survival.

## Conclusions

We feel this study presents unique new findings related to the host response to severe infection in the context of septic shock. Specifically, we found that distinct subgroups of patients can be identified based solely on cytokine profiles and these subgroups show dramatic differences in survival and organ failure. We propose that these subgroups represent subtypes of septic shock. We found that measurement of a small number of cytokines were sufficient to largely reproduce the clinical subgroups defined by a larger panel of 39 signaling molecules. We are not aware of another study which combines a large cohort (363) patients with septic shock with 39 cytokines measured at two well-defined time-points, deep clinical characterization and a sufficient clustering algorithm. In addition, cytokines were not merely reflective of the initial state of illness, as reflected by severity of illness scores such as APACHEII.

Our results contrast with a recent negative report [16] regarding the use of multiplex cytokine measurements which failed to distinguish subgroups of patients from a much broader patient population that also included those with sepsis. This difference may relate to our large sample size in conjunction with more rigorous entry criteria and uniform timing of samples in relation to the onset of disease given the nature of the larger randomized control trial (VASST).

Limitations to this study: Our *post hoc* analysis cannot distinguish causality. Whether cytokine levels are the cause of adverse outcomes or the result of other processes leading to these outcomes is not clear. Our further investigations will attempt to interpret these patterns of immune system signaling compounds in the broader context of known signal transduction pathways and gene expression data, and lead to novel biological hypotheses of pathogenesis of sepsis. We expect that future clinical trials of interventions in sepsis will utilize these results to more clearly characterize subsets of patients that will benefit or face potential harm from interventions.

## Supporting Information

Table S1 Baseline cytokine levels in patient subgroups Low, Medium and High.
(DOCX)

Table S2 24 hour cytokine levels in patient subgroups Low and High.
(DOCX)

Table S3 Features of patients in High subgroup using cytokines at baseline.
(DOCX)

Table S4 Features of patients in High subgroup using cytokines at 24 hours.
(DOCX)

Table S5 Pathway enrichment analysis.
(DOCX)

Table S6 Logistic regression coefficients for predicting patient subgroups.
(DOCX)

Table S7 Patient characteristics by subgroup.
(DOCX)
