# Supplementary material for: Cytokines and Signaling Molecules Predict Clinical Outcomes in Sepsis
Source: PLoS One. 2013 Nov 14;8(11):e79207. doi: 10.1371/journal.pone.0079207 (PMC3828333; doi:10.1371/journal.pone.0079207)
Supplement: Table S1 — Baseline cytokine levels in patient subgroups Low, Medium and High. (DOCX) [file pone.0079207.s001.docx]

**Table S1. Baseline cytokine levels in patient subgroups Low, Medium and High.**

Values are median, and interquartile ranges in pM. Ratio is the ratio of medians of High and Low or Medium cytokine subgroup values. The p-value is from t-test on log10-transformed cytokine values, adjusted for multiple comparison.

| **Cytokine** | **Cytokine Subgroup** | **Patient Subgroup Low** | **Patient Subgroup High** | **Patient Subgroup Medium** | **Ratio Patient Subgroups High to Low** | **p-value (Patient Subgroups Low to High)** | **Ratio Patient Subgroups High to Medium** | **p-value (Patient Subgroups High to Medium)** |
| --- | --- | --- | --- | --- | --- | --- | --- | --- |
| IL6 | 4 | 2 (0.89 - 4.7) | 420 (380 - 430) | 26 (5.4 - 70) | 213.6 | 6.9E-49 | 16 | 2.5E-21 |
| CSF3 | 4 | 3.4 (1.7 - 9.6) | 640 (450 - 950) | 57 (8.4 - 340) | 185.9 | 6.1E-44 | 11.2 | 7.4E-16 |
| IL1RN | 4 | 0.16 (0.031 - 0.7) | 22 (11 - 42) | 4.9 (2.2 - 8) | 135.7 | 2.6E-48 | 4.5 | 3.4E-13 |
| IL10 | 4 | 0.66 (0.31 - 1.7) | 58 (19 - 140) | 4.2 (1.5 - 12) | 87.1 | 1.2E-29 | 13.8 | 1.2E-13 |
| IL9 | 1 | 0.0063 (0.0019 - 0.033) | 0.38 (0.096 - 0.59) | 0.16 (0.095 - 0.3) | 60.1 | 1.8E-19 | 2.4 | 1.5E-01 |
| LTA | 1 | 0.0076 (0.0063 - 0.022) | 0.4 (0.11 - 0.68) | 0.16 (0.082 - 0.33) | 52.6 | 2.1E-29 | 2.5 | 1.2E-03 |
| IL1A | 1 | 0.026 (0.0086 - 0.2) | 1.4 (0.63 - 3) | 0.49 (0.13 - 1.8) | 52.2 | 5.7E-23 | 2.8 | 2.9E-03 |
| IL8 | 4 | 2.7 (1.5 - 4.6) | 140 (34 - 560) | 9.3 (5 - 21) | 50.9 | 8.4E-23 | 14.7 | 9.2E-15 |
| IL2 | 1 | 0.015 (0.0017 - 0.028) | 0.64 (0.43 - 1.1) | 0.2 (0.083 - 0.44) | 41.8 | 3.6E-44 | 3.1 | 2.1E-10 |
| FLT3LG | 1 | 0.025 (0.014 - 0.073) | 0.93 (0.53 - 2.3) | 0.12 (0.025 - 0.39) | 37.7 | 1.0E-22 | 7.7 | 4.9E-13 |
| IL1B | 1 | 0.0088 (0.0043 - 0.016) | 0.28 (0.2 - 0.5) | 0.078 (0.032 - 0.16) | 32.2 | 2.0E-30 | 3.6 | 1.4E-11 |
| IL12B | 1 | 0.036 (0.019 - 0.086) | 1.1 (0.66 - 1.7) | 0.53 (0.19 - 1) | 30.1 | 2.5E-36 | 2 | 9.9E-07 |
| IL13 | 1 | 0.024 (0.012 - 0.086) | 0.66 (0.2 - 1.1) | 0.24 (0.12 - 0.57) | 27.5 | 1.2E-30 | 2.7 | 1.8E-04 |
| CCL2 | 3 | 28 (17 - 54) | 710 (520 - 910) | 120 (56 - 210) | 25.5 | 1.2E-55 | 6.1 | 2.2E-30 |
| CSF2 | 2 | 0.38 (0.21 - 0.63) | 7.5 (5.3 - 12) | 2.2 (1.2 - 3.5) | 19.4 | 5.2E-35 | 3.4 | 3.4E-15 |
| IL4 | 1 | 0.07 (0.022 - 0.17) | 1.1 (0.18 - 1.9) | 0.33 (0.18 - 0.67) | 16.1 | 6.6E-18 | 3.4 | 1.7E-04 |
| CCL7 | 2 | 0.26 (0.17 - 0.6) | 3.8 (2.2 - 6.8) | 1.7 (0.99 - 2.8) | 14.6 | 2.2E-16 | 2.3 | 6.7E-05 |
| CXCL10 | 3 | 40 (19 - 110) | 510 (180 - 1000) | 110 (49 - 400) | 12.8 | 4.1E-15 | 4.6 | 4.1E-05 |
| IL15 | 1 | 0.069 (0.013 - 0.18) | 0.88 (0.52 - 1.4) | 0.44 (0.26 - 0.71) | 12.6 | 8.9E-34 | 2 | 1.1E-07 |
| IL7 | 1 | 0.14 (0.042 - 0.31) | 1.7 (1.3 - 2.4) | 0.69 (0.43 - 1.1) | 11.7 | 1.2E-43 | 2.4 | 3.1E-13 |
| IFNA2 | 2 | 0.33 (0.13 - 0.69) | 3.7 (2.5 - 4.7) | 1.7 (0.93 - 2.7) | 11.4 | 2.6E-33 | 2.2 | 3.6E-07 |
| TNF | 2 | 0.26 (0.16 - 0.46) | 2.9 (1.8 - 5.3) | 0.85 (0.54 - 1.4) | 11.1 | 2.9E-24 | 3.4 | 7.0E-12 |
| TGFA | 1 | 0.03 (0.0088 - 0.059) | 0.29 (0.17 - 0.49) | 0.16 (0.061 - 0.29) | 9.7 | 1.0E-22 | 1.9 | 6.1E-05 |
| CCL3 | 2 | 0.26 (0.099 - 0.52) | 2.5 (1.5 - 4.3) | 0.8 (0.47 - 1.3) | 9.6 | 6.8E-29 | 3.1 | 6.0E-13 |
| IL5 | 1 | 0.02 (0.0038 - 0.048) | 0.17 (0.1 - 0.38) | 0.066 (0.025 - 0.12) | 8.5 | 3.9E-20 | 2.6 | 7.6E-07 |
| IL12P70 | 1 | 0.039 (0.022 - 0.067) | 0.33 (0.21 - 0.45) | 0.17 (0.098 - 0.26) | 8.4 | 8.9E-22 | 1.9 | 9.0E-06 |
| CX3CL1 | 2 | 0.91 (0.35 - 1.6) | 7.3 (4.1 - 9.9) | 3.4 (2.2 - 5) | 8 | 1.1E-20 | 2.2 | 1.3E-06 |
| CXCL1 | 3 | 12 (5.6 - 19) | 80 (43 - 210) | 20 (10 - 36) | 6.8 | 1.9E-18 | 4.1 | 1.4E-11 |
| EGF | 1 | 0.038 (0.02 - 0.086) | 0.25 (0.18 - 0.33) | 0.16 (0.1 - 0.24) | 6.5 | 8.5E-27 | 1.6 | 2.1E-04 |
| VEGFA | 2 | 0.71 (0.39 - 1.1) | 4 (3.1 - 5) | 2.5 (1.7 - 3.4) | 5.7 | 3.3E-29 | 1.6 | 3.9E-06 |
| IL2RA | 4 | 3.7 (1.2 - 8.3) | 20 (11 - 42) | 9.9 (5 - 21) | 5.4 | 1.7E-13 | 2 | 1.1E-02 |
| CCL11 | 4 | 2.0 (1.4 - 3) | 8.9 (6.0 - 14) | 3.9 (2.5 - 5.3) | 4.6 | 1.6E-19 | 2.3 | 2.0E-09 |
| CCL4 | 4 | 2.0 (1.2 - 2.9) | 9.2 (6.8 - 18) | 4.6 (3.3 - 7.1) | 4.5 | 3.5E-21 | 2 | 7.5E-10 |
| IL3 | 1 | 0.036 (0.0097 - 0.077) | 0.16 (0.079 - 0.19) | 0.089 (0.038 - 0.14) | 4.5 | 1.1E-09 | 1.8 | 1.3E-02 |
| FGF2 | 2 | 0.76 (0.41 - 1.1) | 3.2 (2.5 - 5.7) | 2 (1.5 - 2.8) | 4.2 | 2.2E-22 | 1.6 | 3.4E-06 |
| IFNG | 2 | 0.47 (0.28 - 0.78) | 1.8 (1.4 - 3.7) | 1 (0.7 - 1.6) | 3.8 | 2.3E-13 | 1.8 | 2.4E-05 |
| IL17A | 2 | 0.2 (0.1 - 0.32) | 0.71 (0.48 - 2.1) | 0.44 (0.27 - 0.71) | 3.6 | 2.2E-15 | 1.6 | 6.3E-05 |
| CD40LG | 3 | 5.8 (2.4 - 13) | 12 (5.9 - 33) | 12 (5.1 - 24) | 2.2 | 1.7E-04 | 1.1 | 2.6E-01 |
| CCL22 | 3 | 17 (9.6 - 25) | 25 (13 - 47) | 22 (13 - 33) | 1.5 | 2.9E-02 | 1.1 | 8.3E-01 |
