# Supplementary material for: Cytokines and Signaling Molecules Predict Clinical Outcomes in Sepsis
Source: PLoS One. 2013 Nov 14;8(11):e79207. doi: 10.1371/journal.pone.0079207 (PMC3828333; doi:10.1371/journal.pone.0079207)
Supplement: Table S2 — 24 hour cytokine levels in patient subgroups Low and High. (DOCX) [file pone.0079207.s002.docx]

**Table S2. 24 hour cytokine levels in patient subgroups Low and High.**

Values are median, and interquartile ranges in pM. Ratio is the ratio of medians of high and low cytokine subgroup values. The p-value is from t-test on log10-transformed cytokine values, adjusted for multiple comparison.

| **Cytokine** | **Cytokine Cluster** | **High Patient Subgroup** | **Low Patient Subgroup** | **Ratio** | **p-value** |
| --- | --- | --- | --- | --- | --- |
| CCL11 | 3 | 4.3 (3.2 - 7.1) | 1.9 (1.1 - 2.7) | 2.3 | 3.4E-23 |
| CCL2 | 4 | 200 (70 - 560) | 26 (12 - 55) | 7.9 | 9.6E-21 |
| CCL22 | 4 | 18 (11 - 36) | 16 (9.4 - 24) | 1.1 | 3.1E-02 |
| CCL3 | 2 | 1.1 (0.63 - 1.8) | 0.25 (0.099 - 0.51) | 4.5 | 1.2E-28 |
| CCL4 | 3 | 5.2 (3.5 - 7.6) | 2.1 (1.2 - 3.2) | 2.5 | 8.9E-25 |
| CCL7 | 2 | 2.4 (1.4 - 5.4) | 0.26 (0.23 - 0.71) | 9.4 | 1.7E-24 |
| CD40LG | 4 | 9.6 (4.1 - 31) | 6 (2.6 - 18) | 1.6 | 1.7E-03 |
| CSF2 | 2 | 2.8 (1.8 - 5.7) | 0.37 (0.17 - 0.75) | 7.4 | 2.9E-45 |
| CSF3 | 3 | 100 (9.8 - 720) | 3 (1.4 - 8.2) | 35.0 | 9.6E-21 |
| CX3CL1 | 2 | 4 (2.8 - 6.2) | 0.97 (0.18 - 1.7) | 4.1 | 6.3E-37 |
| CXCL1 | 4 | 22 (14 - 59) | 10 (5.4 - 18) | 2.2 | 1.8E-11 |
| CXCL10 | 4 | 300 (85 - 830) | 40 (18 - 98) | 7.4 | 1.9E-18 |
| EGF | 1 | 0.19 (0.13 - 0.27) | 0.043 (0.019 - 0.094) | 4.3 | 9.3E-29 |
| FGF2 | 2 | 2.3 (1.7 - 3.8) | 0.85 (0.35 - 1.3) | 2.7 | 1.3E-27 |
| FLT3LG | 1 | 0.47 (0.068 - 1.1) | 0.025 (0.014 - 0.073) | 19.3 | 1.3E-13 |
| IFNA2 | 2 | 2 (1.3 - 3.4) | 0.28 (0.11 - 0.76) | 6.9 | 3.9E-33 |
| IFNG | 2 | 1.3 (0.77 - 2) | 0.52 (0.32 - 0.79) | 2.6 | 1.5E-16 |
| IL10 | 3 | 9.8 (2.7 - 27) | 0.66 (0.24 - 1.8) | 14.9 | 3.5E-25 |
| IL12B | 1 | 0.74 (0.35 - 1.4) | 0.041 (0.021 - 0.086) | 18.1 | 7.7E-40 |
| IL12P70 | 1 | 0.2 (0.14 - 0.29) | 0.044 (0.022 - 0.076) | 4.6 | 2.8E-29 |
| IL13 | 1 | 0.29 (0.14 - 0.77) | 0.033 (0.013 - 0.11) | 8.8 | 2.6E-32 |
| IL15 | 1 | 0.64 (0.4 - 1) | 0.1 (0.022 - 0.27) | 6.2 | 7.9E-42 |
| IL17A | 2 | 0.49 (0.29 - 0.81) | 0.22 (0.12 - 0.38) | 2.2 | 5.5E-14 |
| IL1A | 1 | 1 (0.44 - 2.4) | 0.033 (0.013 - 0.16) | 31.1 | 5.7E-38 |
| IL1B | 1 | 0.13 (0.074 - 0.29) | 0.0088 (0.0046 - 0.021) | 15.0 | 1.2E-32 |
| IL1RN | 1 | 5.5 (2.4 - 13) | 0.14 (0.03 - 0.66) | 38.9 | 2.8E-53 |
| IL2 | 1 | 0.39 (0.19 - 0.62) | 0.018 (0.0034 - 0.051) | 21.6 | 5.0E-52 |
| IL2RA | 3 | 17 (9 - 33) | 4.4 (1.4 - 9.6) | 3.8 | 1.7E-16 |
| IL3 | 1 | 0.096 (0.045 - 0.15) | 0.04 (0.009 - 0.082) | 2.4 | 3.8E-05 |
| IL4 | 1 | 0.49 (0.18 - 1.2) | 0.072 (0.022 - 0.18) | 6.7 | 3.4E-28 |
| IL5 | 1 | 0.098 (0.052 - 0.16) | 0.024 (0.0075 - 0.046) | 4.0 | 1.1E-13 |
| IL6 | 3 | 20 (6 - 120) | 0.94 (0.21 - 3.7) | 21.8 | 5.2E-22 |
| IL7 | 1 | 0.92 (0.47 - 1.5) | 0.15 (0.048 - 0.39) | 6.1 | 2.8E-29 |
| IL8 | 3 | 12 (3.7 - 48) | 2.1 (0.99 - 4.8) | 5.7 | 1.5E-14 |
| IL9 | 1 | 0.21 (0.096 - 0.41) | 0.0094 (0.002 - 0.051) | 22.2 | 1.9E-28 |
| LTA | 1 | 0.23 (0.11 - 0.46) | 0.012 (0.0063 - 0.05) | 19.6 | 6.7E-41 |
| TGFA | 1 | 0.19 (0.086 - 0.38) | 0.028 (0.0088 - 0.06) | 6.8 | 4.4E-26 |
| TNF | 2 | 1 (0.59 - 1.9) | 0.28 (0.16 - 0.51) | 3.6 | 4.9E-25 |
| VEGFA | 2 | 3.1 (2.2 - 4.1) | 0.83 (0.34 - 1.3) | 3.7 | 9.7E-41 |
