# Supplementary material for: Cytokines and Signaling Molecules Predict Clinical Outcomes in Sepsis
Source: PLoS One. 2013 Nov 14;8(11):e79207. doi: 10.1371/journal.pone.0079207 (PMC3828333; doi:10.1371/journal.pone.0079207)
Supplement: Table S3 — Features of patients in High subgroup using cytokines at baseline. (DOCX) [file pone.0079207.s003.docx]

**Table S3. Features of patients in High subgroup using cytokines at baseline.**

| Feature | p-value^a^ | Enrichment^b %^ |
| --- | --- | --- |
| Blood culture positive^c^ | 0.03 (2.4e-04) | 93 (0.47/0.24) |
| Day 28 death | 0.03 (3.5e-04) | 80 (0.51/0.28) |
| Day 90 death | 0.03 (3.2e-04) | 63 (0.62/0.38) |
| Hematology/Coagulopathy^d^ | <0.01 (2.1e-05) | 121 (0.47/0.21) |
| Immunocompromised | 0.03 (4.8e-04) | 140 (0.3/0.12) |
| Severe septic shock^e^ | <0.01 (1.9e-07) | 59 (0.89/0.56) |

a: p-values represent chance of this enrichment by chance, adjusted for multiple testing using FDR. Values in brackets are not adjusted.

b: Enrichment is the ratio of proportions of patients having this feature value, divided by the overall proportion for all patients. Proportions for this cluster and overall are given in brackets.

c: Blood tested positive for Gram-negative or Gram-positive bacteria

d: Chronic restrictive, obstructive or vascular disease resulting in severe exercise restriction

e: Severe septic shock defined as requiring ≥15 μg/min vasopressors
