# Supplementary material for: Cytokines and Signaling Molecules Predict Clinical Outcomes in Sepsis
Source: PLoS One. 2013 Nov 14;8(11):e79207. doi: 10.1371/journal.pone.0079207 (PMC3828333; doi:10.1371/journal.pone.0079207)
Supplement: Table S4 — Features of patients in High subgroup using cytokines at 24 hours. (DOCX) [file pone.0079207.s004.docx]

**Table S4. Features of patients in High subgroup using cytokines at 24 hours.**

| Feature | p-value^a^ | Enrichment^b^  % |
| --- | --- | --- |
| Blood culture positive^c^ | 0.03 (9.6e-04) | 58 (0.38/0.24) |
| Day 28 death | <0.01 (3.8e-06) | 74 (0.49/0.28) |
| Day 90 death | <0.01 (2.1e-06) | 60 (0.6/0.38) |
| Hematology/Coagulopathy^d^ | 0.01 (3.9e-04) | 69 (0.36/0.21) |
| Immunocompromised | 0.03 (1.1e-03) | 89 (0.23/0.12) |
| Renal failure, 6hr^e^ | 0.03 (9.9e-04) | 66 (0.33/0.2) |
| Renal failure, chronic | 0.04 (1.6e-03) | 21 (0.8/0.66) |
| Sedated at 6 hr | 0.04 (2.1e-03) | 14 (0.91/0.8) |
| Severe septic shock^f^ | <0.01 (1.5e-04) | 32 (0.74/0.56) |

a: p-values represent chance of this enrichment by chance, adjusted for multiple testing using FDR. Values in brackets are not adjusted.

b: Enrichment is the ratio of proportions of patients having this feature value, divided by the overall proportion for all patients. Proportions for this cluster and overall are given in brackets.

c: Blood tested positive for Gram-negative or Gram-positive bacteria

d: New organ failure indicated by platelet count < 80,000/mm^3^

e: Need for renal replacement at 6 hours after admission

f: Severe septic shock defined as requiring ≥15 μg/min vasopressors
