# Supplementary material for: Cytokines and Signaling Molecules Predict Clinical Outcomes in Sepsis
Source: PLoS One. 2013 Nov 14;8(11):e79207. doi: 10.1371/journal.pone.0079207 (PMC3828333; doi:10.1371/journal.pone.0079207)
Supplement: Table S5 — Pathway enrichment analysis. (DOCX) [file pone.0079207.s005.docx]

**Table S5. Pathway enrichment analysis.** Cytokine cluster refers to Figures 7 and 8, numbered from the left.

| Cytokine sample time | KEGGID | KEGG Pathway Name | Cytokine cluster | p-value | Adjusted p-value | Odds ratio | Number of cytokines from cluster found in pathway | Expected number of cytokines from cluster found in pathway | Total number of cytokines found in pathway | Gene symbols in pathway |
| --- | --- | --- | --- | --- | --- | --- | --- | --- | --- | --- |
| Baseline | 4650 | Natural killer cell mediated cytotoxicity | 2 (blue) | 0.003 | 0.18 | Inf | 4 | 1.1 | 4 | CSF2 IFNA2 IFNG TNF |
| Baseline | 4062 | Chemokine signaling pathway | 3 (black) | 0.01 | 0.76 | 17.3 | 4 | 1.4 | 10 | CCL2 CCL22 CXCL1 CXCL10 |
| Baseline | 4940 | Type I diabetes mellitus | 1 (red) | 0.07 | 1.00 | 5.0 | 5 | 2.8 | 7 | IL12B IL1A IL1B IL2 LTA |
| Baseline | 5144 | Malaria | 4 (green) | 0.07 | 1.00 | 6.7 | 4 | 1.7 | 9 | CSF3 IL10 IL6 IL8 |
| 24h | 4650 | Natural killer cell mediated cytotoxicity | 2 (blue) | 0.003 | 0.18 | Inf | 4 | 1.1 | 4 | CSF2 IFNA2 IFNG TNF |
| 24h | 4062 | Chemokine signaling pathway | 4 (black) | 0.01 | 0.76 | 17.3 | 4 | 1.4 | 10 | CCL2 CCL22 CXCL1 CXCL10 |
| 24h | 5144 | Malaria | 3 (green) | 0.04 | 1.00 | 6.7 | 4 | 1.7 | 9 | CSF3 IL10 IL6 IL8 |
| 24h | 4940 | Type I diabetes mellitus | 1 (red) | 0.09 | 1.00 | 5.0 | 5 | 2.8 | 7 | IL12B IL1A IL1B IL2 LTA |
