# Supplementary material for: Cytokines and Signaling Molecules Predict Clinical Outcomes in Sepsis
Source: PLoS One. 2013 Nov 14;8(11):e79207. doi: 10.1371/journal.pone.0079207 (PMC3828333; doi:10.1371/journal.pone.0079207)
Supplement: Table S6 — Logistic regression coefficients for predicting patient subgroups. (DOCX) [file pone.0079207.s006.docx]

**Table S6. Logistic regression coefficients for predicting patient subgroups.** Each set of coefficients define the most complex logistic regression model before additional terms are non significant (p>0.05). Available terms were each of the cytokines and the product IL2 x CSF2. Since only two subgroups are identified for cytokines at 24 hours, the model predicting Low subgroup for 24 hour cytokine data is identical to the High model but with reversed sign on coefficients.

| **Cytokine time** | **Patient subgroup** | **Coefficient** | **Estimate (Std Error)** | **Std. Error** | **p** |
| --- | --- | --- | --- | --- | --- |

| Baseline | Low (AUC=0.99) | Intercept | -7.94 | 1.34 | 3E-09 |
| --- | --- | --- | --- | --- | --- |
| Baseline |  | CSF2 | 4.08E+11 | 1.26E+11 | 0.001 |
| Baseline |  | CCL2 | 5.02E+09 | 1.44E+09 | 0.0005 |
| Baseline |  | IL6 | 8.19E+09 | 2.24E+09 | 0.0003 |
| Baseline |  | TNF | 5.68E+11 | 1.93E+11 | 0.003 |
| Baseline | High (AUC=0.99) | Intercept | -15.1 | 4.2 | 0.000339 |
| Baseline |  | CSF2 | 9.005e+11 | 3.402e+11 | 0.008122 |
| Baseline |  | CCL2 | 6.138e+09 | 2.145e+09 | 0.004218 |
| Baseline |  | IL6 | 1.832e+10 | 5.676e+09 | 0.001244 |
| Baseline |  | TNF | 1.013e+12 | 3.662e+11 | 0.005661 |
| Baseline |  | FLT3LG | 7.700e+11 | 3.786e+11 | 0.041942 |
| Baseline |  | IL9 | -6.734e+12 | 2.032e+12 | 0.000922 |
| Baseline |  | IL2 | 3.504e+12 | 1.270e+12 | 0.005797 |
| Baseline | Medium (AUC=0.88) | (Intercept) | -1.605e+00 | 1.843e-01 | < 2e-16 |
| Baseline |  | LTA | 3.516e+12 | 7.905e+11 | 8.65e-06 |
| Baseline |  | IL2 x CSF2 | -5.700e+23 | 1.010e+23 | 1.68e-08 |
| Baseline |  | IL2 | 3.191e+12 | 7.191e+11 | 9.10e-06 |
| Baseline |  | TGFA | 2.002e+12 | 5.682e+11 | 0.000425 |
| 24 hour | High (AUC=0.99) | Intercept | -2.197e+01 | 6.789e+00 | 0.00121 |
| 24 hour |  | IL2 x CSF2 | 5.785e+24 | 1.821e+24 | 0.00149 |
| 24 hour |  | CSF3 | 7.035e+09 | 2.717e+09 | 0.00961 |
| 24 hour |  | IL4 | 1.617e+13 | 5.414e+12 | 0.00281 |
| 24 hour |  | IL1RN | 1.227e+12 | 4.281e+11 | 0.00416 |
| 24 hour |  | IFNA2 | 3.996e+12 | 1.401e+12 | 0.00433 |
| 24 hour |  | FLT3LG | 6.424e+12 | 2.320e+12 | 0.00562 |
| 24 hour |  | IL8 | 1.578e+11 | 6.076e+10 | 0.00942 |
| 24 hour |  | CCL7 | 6.508e+11 | 3.292e+11 | 0.04808 |
