# Supplementary material for: Cytokines and Signaling Molecules Predict Clinical Outcomes in Sepsis
Source: PLoS One. 2013 Nov 14;8(11):e79207. doi: 10.1371/journal.pone.0079207 (PMC3828333; doi:10.1371/journal.pone.0079207)
Supplement: Table S7 — Patient characteristics by subgroup. (DOCX) [file pone.0079207.s007.docx]

**Table S7. Patient characteristics by subgroup.**

|  | | All | | Baseline | | Baseline | | Baseline | | 24 hours | | 24 hours |
| --- | --- | --- | --- | --- | --- | --- | --- | --- | --- | --- | --- | --- |
|  | |  | | Low | | Medium | | High | | Low | | High |
| Age, years | | 63 (50-72) | | 64 (51-73) | | 63 (50-71) | | 57 (45-74) | | 64 (51-72) | | 62 (47-75) |
| Gender, Females | | 147 (40) | | 72 (38) | | 53 (43) | | 22 (47) | | 112 (40) | | 35 (43) |
| APACHEII score | | 26 (22-32) | | 25 (20-30) | | 28 (23-32) | | 31 (24-34) | | 26 (21-31) | | 29 (24-34) |
| Surgical Diagnosis | | 86 (24) | | 51 (27) | | 29 (23) | | 6 (13) | | 74 (26) | | 12 (15) |
| Severe septic shock | | 204 (56) | | 77 (40) | | 85 (69) | | 42 (89) | | 144 (51) | | 60 (74) |
| Vasopressin treatment | | 188 (52) | | 100 (52) | | 66 (53) | | 22 (47) | | 147 (52) | | 41 (51) |
| Activated Protein C treatment | | 61 (17) | | 20 (10) | | 28 (23) | | 13 (28) | | 41 (15) | | 20 (25) |
| Pre-existing conditions | |  | |  | |  | |  | |  | |  |
| Chronic heart failure | | 73 (20) | | 46 (24) | | 20 (16) | | 7 (15) | | 58 (21) | | 15 (19) |
| Chronic lung disease | | 22 (6.1) | | 20 (10) | | 1 (0.81) | | 1 (2.1) | | 21 (7.4) | | 1 (1.2) |
| Chronic liver disease | | 42 (12) | | 25 (13) | | 14 (11) | | 3 (6.4) | | 32 (11) | | 10 (12) |
| Chronic renal failure | | 241 (66) | | 116 (60) | | 91 (73) | | 34 (72) | | 176 (62) | | 65 (80) |
| COPD | | 60 (17) | | 42 (22) | | 14 (11) | | 4 (8.5) | | 50 (18) | | 10 (12) |
| Cancer | | 68 (19) | | 29 (15) | | 23 (19) | | 16 (34) | | 47 (17) | | 21 (26) |
| Intravenous drug use | | 15 (4.1) | | 8 (4.2) | | 7 (5.6) | | 2 (2-2) | | 13 (4.6) | | 2 (2.5) |
| Alcoholism | | 54 (15) | | 32 (17) | | 19 (15) | | 3 (6.4) | | 46 (16) | | 8 (9.9) |
| Solid organ transplant | | 10 (2.8) | | 7 (3.6) | | 2 (1.6) | | 1 (2.1) | | 7 (2.5) | | 3 (3.7) |
| Recent trauma | | 20 (5.5) | | 13 (6.8) | | 6 (4.8) | | 1 (2.1) | | 15 (5.3) | | 5 (6.2) |
| Immuno-compromised | | 45 (12) | | 17 (8.9) | | 14 (11) | | 14 (30) | | 26 (9.2) | | 19 (23) |
| Chronic steroid use | | 69 (19) | | 39 (20) | | 16 (13) | | 14 (30) | | 46 (16) | | 23 (28) |
| Patient clinical measures at baseline |  | |  | |  | |  | |  | |  | |
| Body temperature (°C) | | 38 (37-38) | | 37 (37-38) | | 38 (37-39) | | 37 (37-38) | | 38 (37-38) | | 38 (37-38) |
| Maximum heart rate (beats per minute) | | 128 (112-140) | | 125 (108-135) | | 130 (116-140) | | 132 (119-144) | | 125 (110-138) | | 134 (120-140) |
| MAP | | 72 (67-78) | | 74 (68-79) | | 71 (66-75) | | 70 (65-74) | | 73 (68-78) | | 71 (65-76) |
| WBC | | 14 ( 7.8-21.1) | | 15 ( 9.9-21.2) | | 14 ( 6.9-22.5) | | 7.1 ( 1.8-10.7) | | 15 ( 9.3-21.1) | | 8.4 ( 3.7-19.2) |
| Platelets | | 167 ( 89-257) | | 195 (109-291) | | 154 ( 94-242) | | 72 ( 49-144) | | 180 (106-290) | | 120 ( 54-191) |
| Pao2Fio2 | | 194 (143-260) | | 206 (158-275) | | 195 (139-230) | | 142 ( 95-208) | | 202 (154-270) | | 162 (112-214) |
| Creatinine | | 155 (44) | | 81 (43) | | 61 (49) | | 13 (29) | | 121 (44) | | 34 (42) |
| Pathogen | |  | |  | |  | |  | |  | |  |
| Gram positive only | | 76 (35) | | 42 (37) | | 21 (30) | | 13 (37) | | 58 (35) | | 18 (35) |
| Gram negative only | | 44 (20) | | 18 (16) | | 15 (22) | | 11 (31) | | 30 (18) | | 14 (27) |
| Fungus only | | 38 (18) | | 23 (20) | | 13 (19) | | 2 (5.7) | | 32 (19) | | 6 (12) |
| Virus only | | 2 (0.92) | | 2 (1.8) | | 0 (0-0) | | 0 (0-0) | | 2 (1.2) | | 0 (0-0) |
| Mixed infection | | 52 (24) | | 28 (25) | | 17 (25) | | 7 (20) | | 40 (24) | | 12 (23) |
| Initial site of infection | |  | |  | |  | |  | |  | |  |
| Lung | | 155 (44) | | 81 (43) | | 61 (49) | | 13 (29) | | 121 (44) | | 34 (42) |
| Intravascular | | 17 (4.8) | | 6 (3.2) | | 6 (4.8) | | 5 (11) | | 10 (3.6) | | 7 (8.8) |
| Abdomen | | 99 (28) | | 53 (28) | | 37 (30) | | 9 (20) | | 84 (30) | | 15 (19) |
| Skin | | 32 (9) | | 19 (10) | | 7 (5.6) | | 6 (13) | | 23 (8.3) | | 9 (11) |
| Genitourinary | | 18 (5.1) | | 8 (4.3) | | 7 (5.6) | | 3 (6.7) | | 14 (5.1) | | 4 (5) |
| CNS | | 2 (0.56) | | 1 (0.53) | | 0 (0-0) | | 1 (2.2) | | 1 (0.36) | | 1 (1.2) |
| Bone/joint | | 5 (1.4) | | 4 (2.1) | | 1 (0.81) | | 0 (0-0) | | 5 (1.8) | | 0 (0-0) |
| Other site | | 28 (7.9) | | 15 (8) | | 5 (4) | | 8 (18) | | 18 (6.5) | | 10 (12) |
